# The Impact of Advances in Fermentation Processes on the Chemical Composition of the Final Product

**DOI:** 10.3390/molecules31040687

**Published:** 2026-02-16

**Authors:** Emilia Janiszewska-Turak, Anna Gramza-Michałowska, Katarzyna Pobiega

**Affiliations:** 1 Department of Food Engineering, Institute of Food Sciences, Warsaw University of Life Sciences—SGGW, Nowoursynowska, 02-776 Warsaw, Poland; 2 Department of Gastronomy Sciences and Functional Foods, Faculty of Food Science and Nutrition, Poznan University of Life Sciences, 60-637 Poznan, Poland; anna.gramza@up.poznan.pl; 3 Department of Biotechnology and Food Microbiology, Institute of Food Sciences, Warsaw University of Life Sciences—SGGW, 02-776 Warsaw, Poland; katarzyna_pobiega@sggw.edu.pl

Fermentation is a complex bioprocess that drives the transformation of raw materials into products with altered chemical makeup, improved nutritional value, and enhanced sensory and functional qualities. Modern fermentation research is increasingly focused on understanding how microbial communities, substrates, and processing conditions interact to steer biochemical pathways toward specific molecular results. This Special Issue of *Molecules*, titled “The Impact of Advances in Fermentation Processes on the Chemical Composition of the Final Product”, features seven original research articles that collectively showcase the versatility of fermentation in both food systems and industrial bioprocessing.

Several contributions highlight the role of fermentation in shaping the chemical composition and functional quality of plant-based products. Lactic acid fermentation has proven to be an effective method for boosting antioxidant capacity, preserving microbial viability, and ensuring chemical stability in freeze-dried fruit matrices [1]. Meanwhile, the use of plant-derived by-products as fermentation additives demonstrates how fermentation processes can be optimized to enhance both chemical and sensory qualities. For example, using fresh pomegranate peel as a sustainable alternative to traditional fermentation additives in Baijiu production led to reduced formation of undesirable compounds, increased antioxidant capacity, and favorable changes in volatile profiles [2]. Together, these studies showcase fermentation as a key tool in adding value to plant materials and by-products while improving the functional qualities of final products.

The influence of fermentation on lipid composition, nutritional quality, and sensory characteristics is further demonstrated in dairy and cereal-based matrices. Fermented dairy products enriched with selected plant additives showed significant changes in fatty acid profiles, lipid quality indices, and mineral levels, highlighting the synergistic effects of fermentation and bioactive-rich ingredients [3]. In cereal systems, fermentation technology and microbial selection notably affect dough chemistry, aroma compound formation, texture, and consumer acceptance. Both low-temperature wheat dough fermentation using mixed cultures of yeasts and lactic acid bacteria and the application of various fermentation techniques in hemp-enriched bread have shown that fermentation conditions play a crucial role in shaping the chemical and sensory profiles of bakery products [4,5].

Fermentation-driven modification of volatile compounds and aroma development emerges as a key theme across various food matrices. In fermented meat products, the addition of tomato pomace affected the formation of volatile compounds and sensory attributes, demonstrating how fermentation can be combined with plant-based ingredients to enhance flavor complexity while promoting more sustainable processing methods [6].

Beyond food applications, this Special Issue also explores industrial fermentation processes, highlighting the wider significance of fermentation-driven chemical changes. Optimization of ethanol fermentation using hydrothermally pretreated cellulose waste revealed that selecting the right pretreatment conditions, fermentation methods, and microbial strains can greatly enhance fermentation efficiency and product yield [7]. This contribution emphasizes the importance of fermentation chemistry in biotech and circular economy contexts.

Overall, the articles in this Special Issue show that advances in fermentation processes greatly impact the chemical makeup, functional properties, and quality of final products across various matrices. By combining microbial selection, substrate innovation, and process optimization, the studies here deepen our understanding of how fermentation can be used as a precise and adaptable tool for chemical, functional, and sensory innovation [1,2,3,4,5,6,7].
